# Global evolution and phylogeography of *Brucella melitensis* strains

**DOI:** 10.1186/s12864-018-4762-2

**Published:** 2018-05-10

**Authors:** Sergey V. Pisarenko, Dmitry A. Kovalev, Anna S. Volynkina, Dmitry G. Ponomarenko, Diana V. Rusanova, Nina V. Zharinova, Anna A. Khachaturova, Lyudmila E. Tokareva, Irina G. Khvoynova, Alexander N. Kulichenko

**Affiliations:** 1 Stavropol Research Anti-Plague Institute, 13-15 Sovetskaya Street, 355035 Stavropol, Russia; 2 Irkutsk Research Anti-Plague Institute, Irkutsk, Russian Federation 664047

**Keywords:** *Brucella melitensis*, Phylogeography, Evolution, Whole genome sequencing, Single nucleotide polymorphism

## Abstract

**Background:**

Brucellosis is a bacterial zoonotic disease. Annually in the world more than 500,000 new cases of brucellosis in humans are registered. In this study, we propose an evolutionary model of the historical distribution of *B. melitensis* based on the full-genomic SNP analysis of 98 strains.

**Results:**

We performed an analysis of the SNP of the complete genomes of 98 *B. melitensis* strains isolated in different geographical regions of the world to obtain relevant information on the population structure, genetic diversity and the evolution history of the species. Using genomic sequences of 21 strains of *B. melitensis* isolated in Russia and WGS data from the NCBI database, it was possible to identify five main genotypes and 13 species genotypes for analysis. Data analysis based on the Bayesian Phylogenetics and Phylogeography method allowed to determine the regions of geographical origin and the expected pathways of distribution of the main lines (genotypes and subgenotypes) of the pathogen.

**Conclusions:**

Within the framework of our study, the model of global evolution and phylogeography of *B. melitensis* strains isolated in various regions of the planet was proposed for the first time. The sets of unique specific SNPs described in our study, for all identified genotypes and subgenotypes, can be used to develop new bacterial typing and identification systems for *B. melitensis*.

**Electronic supplementary material:**

The online version of this article (10.1186/s12864-018-4762-2) contains supplementary material, which is available to authorized users.

## Background

Brucellosis is a particularly dangerous, zoonotic infectious disease caused by bacteria of the genus *Brucella*, which has a high socioeconomic and economic significance [1–3]. The genus *Brucella* is represented by 12 species of microorganisms [4, 5]. The most epidemiological significance is represented by the *B. melitensis* strain - small cattle brucellosis pathogen, causing the most severe forms of the disease [6–8].

A high morbidity level of brucellosis in humans and animals is found in many regions, especially in South America, Africa, the Middle East and much of Asia [9]. In Russia, cases of brucellosis in humans are recorded annually in regions with developed livestock in the South of the European part of Russia and in Siberia [10–13].

To date, the gold standard of molecular typing of of the genus *Brucella* reprecentatives is considered to be multi-locus VNTR-analysis (MLVA) [14]. A number of studies have demonstrated that MLVA-16 is an effective tool for genotyping when conducting epidemiological investigations of brucellosis outbreaks [15, 16]. However, with the advent of the SNP analysis of complete genomes, intraspecies differentiation and the establishment of the origin of individual strains of the brucellosis pathogen have reached a qualitatively new level [17].

Based on the analysis of the genome sequences at the loci of single nucleotide polymorphisms (SNP) localized in the orthologous genes of *B. melitensis* strains, Kim-Kee Tan et al. draw a map of the global genetic diversity of *B. melitensis* strains isolated on the territory of different continents [18]. In a study published by the authors, *B. melitensis* isolates are represented by five genotypes. Mediterranean strains are identified as genotype I, Asian strains are classified as genotype II, genotype III is represented by strains of African descent. The genotypes IV and V are assigned respectively to the European and American lines. Earlier, we gave a phylogeographic characterization of strains of *B. melitensis* isolated in the Russian Federation in the North Caucasus [19], which form a separate cluster belonging to genotype II.

The aim of our study was to study the phylogenetics of the *B. melitensis* starins that has been previously exposed and isolated on different continents. In this paper, we propose a possible historical reconstruction of the distribution of the *B. melitensis* starins all over the world. We present a phylogeny reconstruction based on Bayesian analysis of the full genome SNP of 98 *B. melitensis* strains. The results of our study may help to contribute to a better understanding of the epidemiology and spread of *B. melitensis*.

## Results

### General results

Genomic sequences of 11 *B. melitensis* isolates isolated collected in Russia in Siberia and the South of the European part of Russia. They were generated by high-performance sequencing using the platform Ion Torrent PGM (Life Technologies, USA). The resulting reads were de novo assembled in unclosed draft genomes. Annotation of draft genomes was carried out with the help of NCBI Prokaryotic Genome Annotation Pipeline. The obtained genomic projects were deposited in the GenBank database. General characteristic of genomes is presented in Additional file 1: Table S1.

We used the genomic sequence of 98 strains of *B. melitensis*, eleven isolates were obtained while this study and 87 genomic sequences were taken from the international database GenBank. We used all the complete genomes and WGS projects that were available at the time of our research. Previously, a close phylogenetic relationship between *B. melitensis* and *B. abortus* species was described [20], which determined the choice of genomic sequence of *B. abortus* 2308 strain as an external group. Information on the genomic sequences of strains used in the work is presented in Additional file 2: Table S2. Information on the genomic sequences of strains used in the work is presented in Additional file 2: Table S2.

To build a multiple alignment matrix of 99 genomes of *Brucella* strains we used REALPHY 1.10 with default settings [21]. Using algorithms implemented in REALPHY allowed us to obtain a matrix of multiple genome alignment containing only orthologous nucleotide sequences - so-called core genome. All the similar sequences were excluded from multiple alignments. The resulting multiple alignment matrix of complete genomes was used to build the phylogeny of *B. melitensis*. Phylogenetic reconstruction was carried out using the software package BEAST 2.3.0 [22].

**Fig. 1:**
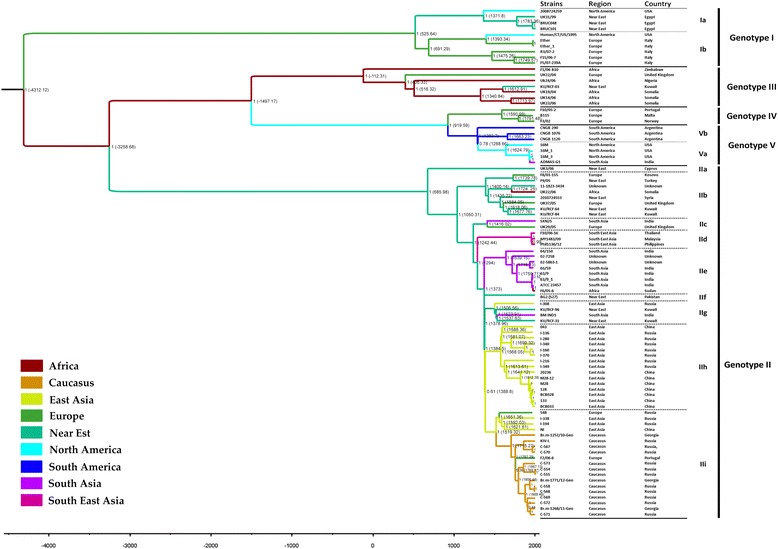
The Bayesian phylogenetic tree shows the evolutionary relationships of the 88 *B. melitensis* isolates (those for which a fixed isolation date was established). The timeline indicates the date of divergence of the branches of the tree, the values of < 0 correspond to the years BC, the ends of the branches of the tree - the isolation time of the strains. The tree nodes indicate the probabilities and Bayesian estimation of time of divergence. The color of the terminal branches of the tree corresponds to the region in which the isolates were isolated

**Fig. 2:**
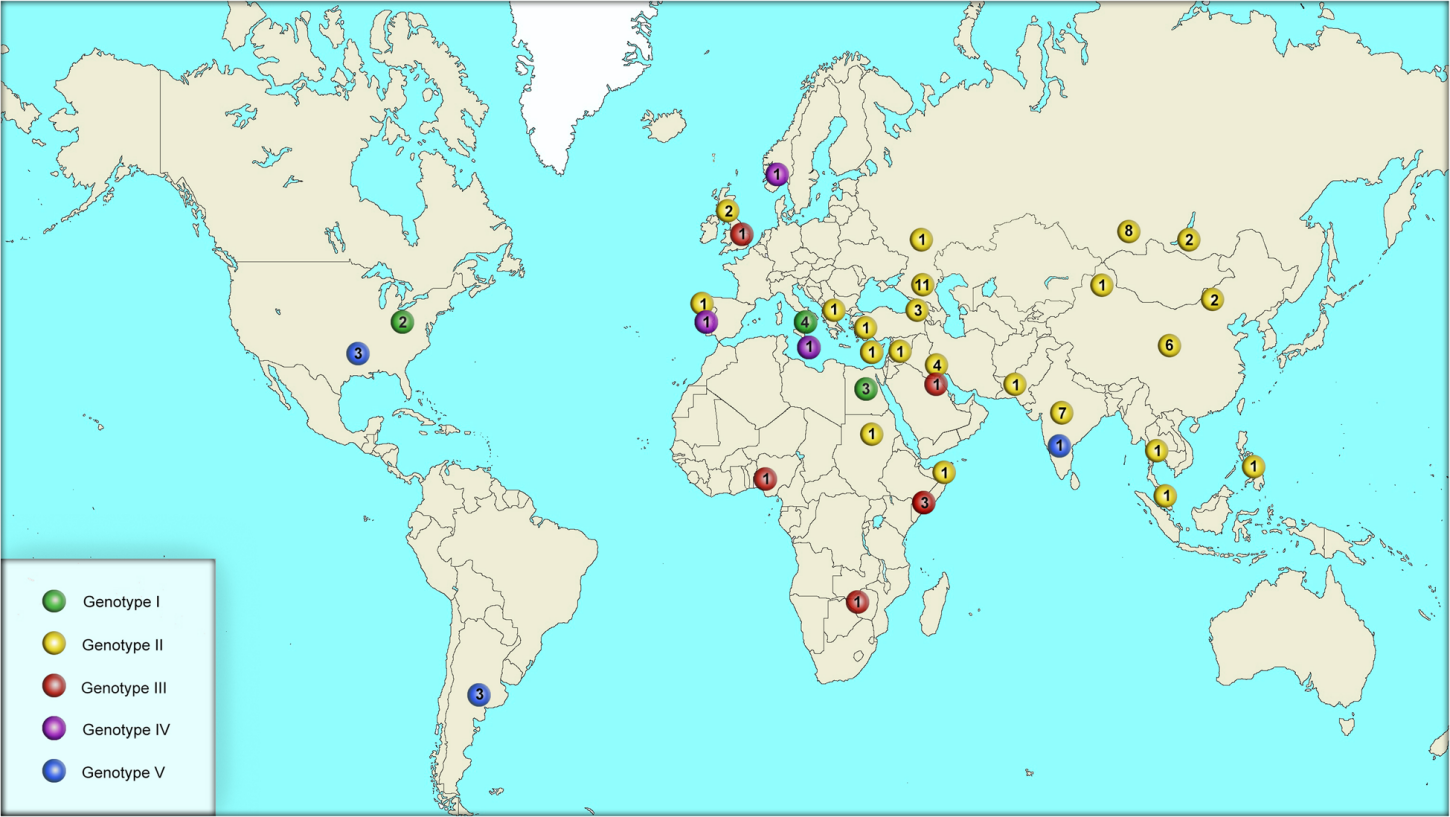
Geographical distribution of isolates. The labels indicate the geographical regions in which the strains were isolated. The color of the label corresponds to the genotype that we determined for each isolate, the number inside the label corresponds to the number of strains isolated in each region

As a result of the phylogenetic reconstruction using the multiple alignment matrix of 99 genomes of *Brucella* strains, two strains of *B. melitensis* S66 and *B. melitensis* 16 M13 W were not related to the species *B. melitensis*. This fact has already been described previously by Kim-Kee Tan et al. [18]. We have also noticed that in these strains the number of SNPs detected in the matrix of the cortical genome (according the matrix of multiple alignment of 99 genomes) is several times higher than the number of SNPs in other strains of the sample, Additional file 2: Table S2. According to the literature data, *B. melitensis* S66 strain was isolated from the blood of a man in Jilin province [23]. *B. melitensis* 16 M13 W strain was isolated from BALB mice after infection for 13 weeks, compared to the 16 M strain used to infect the animal model [24]. It is likely that these strains do not belong to the species *B. melitensis* [18], but we did not have the opportunity to test this hypothesis. Thus, we decided to exclude these two strains sequences from the matrix of the cortical genome. In addition, we removed from the matrix of the cortical genome the sequences of all laboratory and vaccine strains in order to eliminate any possible effect of artificially acquired mutations on the results of the analysis. We also had to exclude the strains where we could not determine the date of exposition, since the use of the wrong date of exposition leads to significant errors in the dating of the phylogenetic tree. All the further manipulations, including phylogenetic and evocative analysis, as well as SNP search, were conducted using the cortical genome matrix, which contained sequences of 88 strains. A phylogenetic tree describing the evolutionary relationships of natural strains is shown in Fig. 1. The geographical map of genotypes with indication of places of isolation of strains is given in Fig. 2.

### Phylogenetic analysis

*B. melitensis* strains used in the study are represented by five main genotypes that correspond to the potential geographical origin of the isolates. *B. melitensis* Ether (biovar 3) was basal to the phylogenetic tree, *B. melitensis* 16 M (biovar 1) and *B. melitensis* 63/9 (biovar 2), were separated in after divergence from *B. melitensis* Ether strain, demonstrate the previously described topology phylogenetic tree [18, 21]. A group of strains including *B. melitensis* Ether we denoted as genotype I, *B. melitensis* 63/9 and related strains - genotype II, strains of the African group formed genotype III, a group of strains, including *B. melitensis* B115 - genotype IV and a clade of *B. melitensis* 16 M strain form the genotype V.

Genotype I represents the most basal lineage of *B. melitensis* strains, and its diversification took place, probably, about 6.5 thousand years ago in the Neolithic period (5-3 thousand years BC). Comparison of genomic sequences of strains of genotype I with the sequence of *B. melitensis* 16 M reference genome allowed to reveal from 2770 to 2886 SNP for each strain. We have found 1237 specific SNPs for strains of this genotype. A pairwise comparison of genomic sequences of strains within genotype I made it possible to identify 1384 SNPs. Genotype I includes two subgenotypes. The subgenotype Ia forms strains predominantly collected on the territory of Egypt, and we have identified 200 SNPs specific for the subgenotype Ia. The comparison of genomes inside the subgenotype allowed us to identify 341 SNPs. Subgenotype Ib predominantly consists of strains isolated in Italy. We have identified 30 SNPs specific for subgenotype Ib. A pairwise comparison of the genomes inside the clade allowed to distinguish 812 SNPs.

Genotype II includes 61 strains of *B. melitensis*. The diversification of this genotype might take place in the second half of the third millennium BC, approximately 5270 years ago. The full-genomic SNP analysis of isolates of *B. melitensis* genotype II revealed from 2271 to 2493 polymorphisms in comparison with the reference genome *B. melitensis* 16 M. The genotype II includes nine subgenotypes, denoted as IIa-IIi.

Subgenotype IIa is represented by a single strain - *B. melitensis* UK3/06. The divergence of this genotype might occur in the second half of the VII century AD. *B. melitensis* UK3/06 strain has 238 unique SNPs that distinguish it from all other strains of the species.

Diversification of the subgenotype IIb, might take place in the middle of the XI century AD. For the strains of this subgenotype, we have identified 86 clade-specific SNPs. A pairwise comparison of the genomes inside the clade allowed to distinguish 771 SNPs.

Subgenotype IIc occupies the internal node on the phylogenetic tree and its divergence might take place in the middle of the XIII century. We have identified 21 clade-specific SNPs for strains of this subgenotype. A pairwise comparison of the genomes inside the clade allowed to distinguish 200 SNPs.

Deviation of the subgenotype II d probably took place at the end of XIII century. We found 128 SNPs of clade-specific strains of this subgenotype. A pairwise comparison of the genomes inside the clade allowed to distinguish 24 SNPs.

Diversification of the subgenotypes IIe, IIf, IIg, IIh and IIi might take place in the second half of the XIV century A.D. For strains of the subgenotype IIe we have found 44 clade-specific SNPs. A pairwise comparison of the genomes inside the clade allowed to distinguish 247 SNPs. The subgenotype IIf is represented by one strain of BG2 (S27) for which we have found 116 unique SNPs. For strains belonging to the subgenotype IIg, we have identify 46 clade-specific SNPs. A pairwise comparison of the genomes inside the clade allowed to distinguish 509 SNPs. Subenotype IIh includes 15 strains for which we have found 41 clade-specific SNPs. A pairwise comparison of the genomes inside the clade allowed to distinguish 640 SNPs. For the strains of the subgenotype IIi we have identified 20 clade-specific SNPs. A pairwise comparison of the genomes inside the clade has shown 789 SNPs.

The divergence of the genotype III might take place in the middle of the II millennium B.C. about 3500 years ago. The genome of the strains belonging to genotype III, total from 1625 to 1771 SNPs when compared to the genome *B. melitensis* 16 M. We have found 330 genotype-specific SNPs for this genotype. A pairwise comparison of the genomes has distinguished 1900 SNPs.

Deviation of genotypes IV and V from a common ancestor might have happen more than 3500 years ago. For genotype IV strains, we were able to detect 122 genotype-specific SNPs. A pairwise comparison of the genomes has distinguished 203 SNPs. Compared with reference genome, the strains of this genotype have from 577 to 607 SNPs.

We have found 82 genotype-specific SNPs for the strains of genotype V. The comparison of the strains with reference genome revealed from 188 to 424 SNPs. A pairwise comparison of strains of genotype V have revealed 615 SNPs. Genotype V is represented by two genotypes which deviation from the common ancestor might occur about 1000 years ago. For the subgenotype Va strains we have found 115 clade-specific SNPs. A pairwise comparison of strains has shown 210 SNPs. For the subgenotype Vb strains, we have not been able to find any clade-specific SNPs. A pairwise comparison of strains of Vb subgenotype has distinguished 288 SNPs.

## Discussion

SNP analysis of the core genome of 88 isolates of *B. melitensis* type helped to describe five genotypes. Using the data on the time of strain isolates, the phylogenetic tree was constructed and the hypothetical time limits for the diversification of the *B. melitensis* genotypes. In addition, we have built a global phylogeographic model of the most pathogenic species for humans - *B. melitensis*.

It seems highly probable that the spread of *B. melitensis* began from the region of the Fertile Crescent, which was one of the centers of the Neolithic. In this region, according to historians, small cattle were domesticated [25], which is the main host for *B. melitensis* bacteria.

The basal of the phylogenetic tree are the strains of genotype I, which diversification might take place 6.5 thousand years ago in the Neolithic period (5-3 thousand years BC). Genotype I is represented by two subgenotypes. The subgenotype Ia - is the strain, isolated in Italy, and subgenotype Ib, − strains, isolated in Egypt. Probably, 6.5 thousand years ago the expansion of cattle-breeding races from the Middle East, where plant- and cattle breeding were developed higher, to the Eastern Europe [26] caused the diversification of genotype I. The deviation of the subgenotypes Ia and Ib from the common ancestor might happen in the middle of the first Millennium A.D. About VI century A.D., the strains of this genotype were transmitted to the territory of the North Africa (Egypt), that possibly contributed to the diversification subgenotype Ib.

The deviation of the second branch of the tree and the formation of genotype II occurred in the second half of the third millennium BC., approximately 5270 years ago. The strains of genotype II have the widest geographical distribution, their habitat is the most of the territory of the Eurasian continent - from Cyprus in the west, to the islands of Southeast Asia in the east. Ladder-like phylogram of the genotype II suggesting a possible single introduction of Mediterranean origin. Dating of phylogenetic tree allows you to determine the approximate date - the end of VII century. The current representative of this branch is *B. melitensis* strain UK3/06, isolated in Cyprus in 2006. It can be assumed that the distribution of the genotype II strains from west to east may be caused by the Arab Caliphate’s conquest of huge territories, from the Mediterranean Sea in the west to the borders of India and China in the east at the end of the first millennium A.D. [27]. The opportunity to control of the most important land routes connecting Eastern Europe - through Central Asia or the Caucasus and the Iranian highlands - with India on the western part of the Great Silk Road provided the Arabs a key role in Europe’s trade with all of South, Southeast Asia, and China [28]. This hypothesis is confirmed by the geographical distribution of the strains of the genotype IIb that were isolated in Turkey, Kuwait and Somalia. The strain *B. melitensis* 2,010,724,553 was isolated in California, USA in 2010 from a patient from Syria who had chronic brucellosis for about 20 years. It is likely that the *B. melitensis* strain F8/01-155, isolated in Kosovo in 2001, and the *B. melitensis* strain UK37/05, isolated in the UK in 2005, are also associated with the importation of the *B. melitensis* into European countries from the Middle East. Unfortunately, we were not able to obtain reliable information on the location and time of isolation of of the strain *B. melitensis* 11-1823-3434, which undoubtedly belongs to the subgenotype IIb.

The further distribution of genotype II strains across the territory of Asia took place on the period of existence and disintegration of the Mongolian Empire in1206-1368. Probably, in the middle of the XIII century A.D. the subgenotype IIc diversificated, with its strains from India and Great Britain. Deviation of the subgenotype IId from the common ancestor took place at the end of the XIII century. The strains of this subgenotype are currently existing in the islands of Southeast Asia. The high degree of affinity of the subgenotype IId strains is confirmed by the relatively small amount of SNPs (24 polymorphisms) that we have found while pairwise comparison of strains within the subgenotype. The topology of the tree makes it possible to assume that the strains of the subgenotype IId were transmitted to the islands by sea from the coast of India. The coastal regions of the Indian peninsula have long tradition of maritime trade with the countries of the Middle East and Southeast Asia [29].

A century later, in a short period of time, in the 70-90s of the XIV century, the diversification of five subgenotypes took place: IIe, IIf, IIg, IIh, IIi. The subgenotype IIe is mostly consists of strains isolated in India. The subgenotype IIf is represented in our study by one strain isolated in Pakistan. Strains of the subgenotype IIg, two of which were isolated in Kuwait, one in Siberia, Russia and one in northern India, form a clearly differentiated group of genetic affinity, obviously with a single geographic region of origin. We assume that the isolates of the subgenotype IIg exists in the northern part of India on the border with Pakistan, and the distribution of representatives of this subgenotype representatives northwards to Russia and westwards to Kuwait might happen in the Middle Ages via the land trade routes. However, to confirm this hypothesis, it is necessary to investigate a larger number of strains of the subgenotype IIg.

Deviation of the subgenotype IIh from the ancestral branch could take place in the XVI century. It is represented by 15 strains, eight of them were isolated in China and seven were isolated in Russia in Eastern Siberia. Two groups of strains forming the subgenotype IIh do not have a clear differentiation according to their teroritritorial affiliation. The first group is formed mainly by strains from Russia, the second - mainly strains from China. The absence of a clear differentiation according to territorial affiliation between these groups assumes the frequent penetration of the *B. melitensis* strains from one country to another. Active trade between Russia and China could promote this process. The most probable way of bringing the *B. melitensis* to the territory of Siberia is the Tea Way - the trade route in the XVI - XIX centuries between Beijing and Moscow, the second route after the Great Silk Road in terms of trade turnover. A pairwise comparison of the strain genomes of this subgenotype made it possible to define 640 SNPs. A pairwise comparison inside the each group allowed to distinguish 366 SNPs for the “Russian” group and 267 SNPs for the “Chinese” group. The low frequency of SNPs within each group indicates a high homogeneity of the strains.

The period of deviation of the subgenotype IIi may be defined as the XVI century A.D. This subgenotype is represented by 19 strains, 14 of them were isolated in Russia. On the phylogenetic tree subgenotype IIi is represented by two clades. The first clade consists of *B. melitensis* 548, *B. melitensis* I-338, *B. melitensis* I-194 and *B. melitensis* NI isolated in Saratov, Novosibirsk region, Irkutsk region and Inner Mongolia (China), respectively. The second clade consists of strains existing in the territory of Kalmykia, the North Caucasus and Georgia, as well as one strain isolated in Portugal, which, according to our information, was brought to Portugal from the North Caucasus. Probably, the spread of the *B. melitensis* subgenotype IIi from the Central Asian countries to the territory of Russia took place via the northern route of the Great Silk Way, that connected the eastern countries with the Northern Europe. From the Lower Volga region the strains of the subgenotype IIi spread to Eastern Siberia, as well as to Kalmykia, the North Caucasus and Transcaucasia. Nucleotide analysis of the core genome of the subgenotype strains has revealed 789 SNPs. A pairwise comparison of the genomes of the different clades allowed to distinguish 354 SNPs in the first clade and 404 SNPs in the second clade.

The divergence of *B. melitensis* strains existing in the Middle East might take place in the middle of the second millennium B.C., about 3500 years ago, due to the genotype III deviation, its modern representatives are isolated in African countries currently. Probably, genotype III strains had previously circulated in North Africa, but global climate changes that caused the desertification of huge areas in the northern part of the continent, could cause migration of cattle-breeding races to the south, to Central Africa, which, in turn, the spread of the *B. melitensis* to new territories. An alternative way of the *B. melitensis* could pass through the Arabian Peninsula and the Bab el Mandeb Strait to East and Central Africa.

The strains of genotype IV, isolated on the territory of European states, probably originate from genotype III and, probably, and might be brought to the territory of Europe from the coast of North Africa. Genotype IV is the smallest among *B. melitensis* genotypes in our study, it included only three strains. Apparently, this is due to the fact that the countries of Northern and Western Europe have achieved almost complete elimination of this disease among farm animals.

Diversification of genotype V, according to our analysis, might take place in the X century A.D. This genotype is represented by strains from the North and South America, except for the *B. melitensis* strain ADMAS-G1, isolated in India. Up to date, the generally recognized pre-Columbian contacts of America and Europe (not considering the primary settlement of America through Beringia in the primitive epoch) are only Viking floats, dating back to around 1000 A.D. and continuing, in all probability, until the XIV century [30–33]. American strains are represented by two subgenotypes. The subgenotype Va consists of strains isolated in North America and India, the subgenotype Vb is formed by three strains from Argentina. It must be noted that we were not able to identify any clade-specific SNPs from South America strains.

We defined polymorphisms specific for the strains of each of the genotypes and subgenotypes. Data on these mutations can be used as markers in the development of new systems for identifying the geographical region of *B. melitensis* strains origin. In 2017, Kim-Kee Tan et al. published the results of the SNP analysis of the nucleotide sequence of the *rpoB* gene to determine the origin of *B. melitensis* strains [34]. In the long term, this approach can be adapted to study representatives of other epidemic-significant *Brucella* species.

The results of our studies confirm that full genomic sequencing (WGS) is an indispensable analysis tool for determining the geographic region of strains origin. The method provides a higher resolution than other PCR-based approaches (MLVA, multilocus SNP typing), but requires reliable and complete metadata. The use of a sufficient amount of verified metadata will allow qualitatively to increase the resolution of the method to the level of individual regions of the country (subject, locality, etc.). Expanding knowledge of the unique features of isolates isolated from different countries and integrating the data into WGS databases will allow for more qualitative use of full genome sequencing to identify the level of genetic linkages between different strains, determine the geographical region of the infection source origin, establish the causes, conditions of animals and people diseases occurrence with brucellosis and localization of the infection focus.

## Conclusion

The SNP analysis of the genomic sequences of *B. melitensis* strains isolated in different geographical regions of the planet confirmed the existence of five genotypes. The results of the study revealed a high degree of similarity of the genome SNP profiles of the *B. melitensis* strains circulating in one area, which makes it possible to consider the method of full genomic SNP analysis as an effective tool for determining the origin of individual isolates in epidemiological investigations.

The paper describes a possible historical reconstruction of *B. melitensis*. Distribution around the world. Strains of *B. melitensis* genotypes existing in Russia are described. The hypothesis about the ways of penetration and further spread of *B. melitensis* in Russia is proposed.

A complex of SNP specific for different genotypes of the *B. melitensis* can be further used in the development of new schemes of *B. melitensis* subgenotypes typing.

## Methods

### Bacterial strains

All strains used in the study were isolated from humans. Strains *B. melitensis* I-136, *B. melitensis* I-160, *B. melitensis* I-194, *B. melitensis* I-216, *B. melitensis* I-280, *B. melitensis* I-308, *B. melitensis* I-338, *B. melitensis* I-340, *B. melitensis* I-349, *B. melitensis* I-380 were transferred from the Irkutsk Anti-Plague Research Institute of Rospotrebnadzor. The *B. melitensis* KIV-L strain was obtained from the State Collection of Pathogenic Microorganisms and Cell Cultures in Obolensk. Isolates were identified and approved by using standard biochemical methods “Epidemiological surveillance and laboratory diagnostics of brucellosis” (MUK 3.1.7.3402-16). Detailed information on the biochemical properties of isolates is given in Additional file 3: Table S3.

### Antibiotic susceptibility testing

Antibiotic susceptibility was determined by disk diffusion on Mueller-Hinton (MH) agar.

in accordance with the guidelines of the Russian protocols of “Epidemiological surveillance and laboratory diagnostics of brucellosis” (MUK 3.1.7.3402-16) at the Stavropol Anti-Plague Control Research Institute. The following commercially available antimicrobial drugs (Research Center of Pharmacotherapy, St. Petersburg, Russia) were tested: amikacin, tetracycline, kanamycin, gentamicin, ciprofloxacin, streptomycin, ofloxacin, pefloxacin, levofloxacin, rifampicin. In Additional file 4: Table S4, for each antibiotic, the diameter of the growth inhibition zone in millimeters is indicated.

### Preparation of genomic DNA samples

Bacteria were cultured on brucella agar at 37 °C for 48 h. The microbial suspension of 2 × 109 m.sub./ml concentration was decontaminated by addition of sodium mertiolate to a final concentration of 0.01% and subsequent incubation at 56 °C. for 30 min. Genomic DNA was isolated from 0.5 ml of a disinfected microbial suspension using the PureLink Genomic DNA Kit (Life Technologies, USA). Concentration of genomic DNA was determined using a Qubit2.0 and Qubit dsDNA HS Assay Kit fluorometer (Invitrogen, Life Technologies, USA). The purity of the genomic DNA was evaluated with a NanoDrop 2000 spectrophotometer (Thermo Scientific, USA). Sample preparation was carried out in the laboratory of brucellosis research of the Stavropol Anti-Plague Institute of Rospotrebnadzor.

### DNA library preparation and whole genome sequencing

Preparation of genomic libraries with a length of ridges 400 b.p. was used with Ion XpressTM Plus Fragment Library Kit (Life Technologies, USA) in accordance with the manufacturer’s protocol. Fragments of DNA libraries were separated using 2% agarose gel E-Gel SizeSelect (Invitrogen, USA). The prepared libraries of DNA fragments were purified using Agencourt AMPure XP (Beckman Coulter, USA). The quality and concentration of the libraries was determined with using the Experion ™ Automated Electrophoresis System and Experion DNA 1 K Reagents and Supplies and Experion DNA Chips (Bio-Rad, USA). Monoclonal amplification on microspheres was performed with using Ion OneTouch 400 Template Kit (Life Technologies, USA) reagent kits according to the manufacturer’s protocol. Microspheres were enriched with magnetic particles Dynabeads MyOne Streptavidin C1 (Invitrogen, Life Technologies, USA). The efficiency of the enrichment process was evaluated using the Ion Sphere Quality Control Kit (Life Technologies, USA). Genome sequencing was performed using the Ion Torrent PGM sequencer and Ion 318 Chips Kit V2 (Life Technologies, USA).

### Post-sequencing data processing

Evaluation of the quality of the received reides was carried out using the FastQC program version 0.11.3 [35]. Reids containing nucleotides with quality of reading Q < 15 were delited in Trimmomatic version 0.33 [36]. Reids with an average quality of Q < 20, as well as reids less than 75 nucleotides long were removed. The genome was assembled in the software Newbler v 3.0 (Roche). Evaluation of the quality of genome assembly was performed using the Quast 3.0 program [37], the genomic sequence of *B. melitensis* 16 M strain (GeneBank: NC003317, NC003318) was used as a reference to evaluate the accuracy and efficiency of assembly of genomic projects. Annotation of genomic projects was carried out with the help of NCBI Prokaryotic Genome Annotation Pipeline (PGAAP).

### Whole genome SNP analysis

To build cortical genome we used 11 *B. melitensis* genomes sequenced by us, 87 *B. melitensis* genomes that are publicly available, including complete genomes and genome projects and the *B. abortus* 2308 genome as external groups. These sequences were submitted to the Reference Sequence Alignment-based Phylogeny Builder (REALPHY) [21] for the identification of sites that were relevant for the phylogenomic analysis using the default parameters. The complete genome of *B. melitensis* 16 M was chosen as the reference genome, and all of the query genomic sequences were divided into possible sequences of 50 bp (default) and subsequently mapped to the reference genome via Bowtie2 with a default k-mer length of 21, allowing for one mismatch within the k-mers to maximise sensitivity. The REALPHY method has two standard thresholds for trusting the base call. The first is that the weight of the mapping has to be higher than 10, the second is that 95% of the mappings has to support the same nucleotide.

Using algorithms implemented in REALPHY allows to obtain a matrix of multiple genome alignment containing only homologous nucleotide sequences – the so-called core genome. Editing of the multiple alignment matrix (the reasons are given in the Results section) and SNP identification with the formation of the base of nucleotide polymorphisms sites were performed in the MEGA 7 program [38]. The base of sites of nucleotide polymorphisms of strains of *Brucella* is found in the supplementary file, Additional file 5: Table S5.

### Phylogeographical and evolutionary analysis

For studying the phylogeographic distribution of taxa on the basis of the full genome analysis of SNP *B. melitensis* strains, we used the BEAST 2.3.0 software package [22]. For the dating of the phylogenetic tree, the known isolation dates of *B. melitensis* strains were used. The parameters of the evolutionary model were determined by estimating the work of 88 different substitution models on the matrix of the genomes multiple alignment using the Jmodeltest2 program [39, 40]. The following model parameters were defined: priority model GTR + I + G, rateAC = 0.972, rateAG = 3.369, rateAT = 0.496, rateCG = 0.555, rateGT = 0.995, proportion Invariant = 0.733. We carried out three independent runs using strict clock, a long chain of 200,000,000 for each run, and a recording rate of every 1000 generations. An estimate of the convergence of the MCMC topology and parameters was carried out in the Tracer v1.6 program [41]. All ESS parameter values were > 200. The trees were combined using the TreeAnnotator component from the BEAST 2.3.0 package to obtain a consensus tree, the burn-in parameter for each chain was defined as 20%, the resulting phylogenetic tree file was visualized in the Figtree program [42].

## Additional Files

Additional file 1: **Table S1.** Characteristics of genomic projects. (DOCX 17 kb)

Additional file 2: **Table S2.** List of *B. melitensis* genomes used in this study. (DOCX 34 kb)

Additional file 3: **Table S3.** Biochemical properties of isolates. (DOCX 18 kb)

Additional file 4: **Table S4.** Antibiotic susceptibility testing. (DOCX 16 kb)

Additional file 5: **Table S5.** Base of sites of nucleotide polymorphisms of *Brucella* strains. (XLSX 19468 kb)

## Abbreviations

MLVA: Multiple Loci VNTR Analysis
SNP: Single Nucleotide Polymorphism
VNTR: Variable Number Tandem Repeat
WGS: Whole Genome Sequencing

## References

[1] Plumb GE, Olsen SC, Buttke D (2013). Brucellosis: ‘one health’ challenges and opportunities. Rev Sci Tech Off Int Epiz.

[2] Mantur BG, Mulimani MS, Bidari LH (2008). Bacteremia is as unpredictable as clinical manifestations in human brucellosis. Int J Infect Dis.

[3] Nicoletty P (2010). Brucellosis: past, present and future. Sec Biol Med Sci.

[4] Whatmore AM, Davison N, Cloeckaert A, Al Dahouk S, Zygmunt MS, Brew SD (2014). *Brucella papionis* sp. nov., isolated from baboons (Papio spp.). Int J Syst Evol Microbiol.

[5] Scholz HC, Revilla-Fernández S, Al Dahouk S, Hammerl JA, Zygmunt MS, Cloeckaert A (2016). *Brucella vulpis* sp. nov., isolated from mandibular lymph nodes of red foxes (Vulpes vulpes). Int J Syst Evol Microbiol.

[6] Franz DR, Parrott CD, Takafuji ET, Sidell FR, Takafuji ET, Franz DR (1997). The US biological warfare and biological defense programs. Medical aspects of chemical and biological warfare.

[7] Kaufmann AF, Meltzer MI, Schmid GP (1997). The economic impact of a bioterrorist attack: are prevention and postattack intervention programs justifiable?. Emerg Infect Dis.

[8] Pappas G, Panagopolou P, Christou L, Akritidis N (2006). *Brucella* as a biological weapons. Cell Mol Life Sci.

[9] Pappas G, Papadimitriou P, Akritidis N, Christou L, Tsianos EV (2006). The new global map of human brucellosis. Lancet Infect Dis.

[10] Lyamkin GI, Tikhenko NI, Manin EA, Vilinskaya SV, Golovnev SI, Rusanov DV, Kulichenko AN (2011). Epizootiological and epidemiological situation on brucellosis in the Russian Federation in 2010 and prognosis for 2011. Probl. Osobo Opasn. Infek..

[11] Lyamkin GI, Tikhenko NI, Manin EA, Rusanova DV, Golovneva SI, Vilinskaya SV, Kulichenko AN (2012). Brucellosis epidemiological situation and its morbidity in the Russian Federation in 2011, and prognosis for 2012. Probl Osobo Opasn Infek.

[12] Lyamkin GI, Manin EA, Golovneva SI, Tikhenko NI, Kulichenko AN (2013). Epidemiologic situation on brucellosis in the Russian Federation in 2012 and prognosis for 2013. Probl. Osobo Opasn. Infek..

[13] Lyamkin GI, Golovneva SI, Khudoleev AA, Chebotareva EN, Shakirova LI, Kulichenko AN (2014). Survey of epizootiologic and epidemiologic situation on brucellosis in the Russian Federation in 2013; prognosis for 2014. Probl. Osobo Opasn. Infek..

[14] Scholz HC, Vergnaud G (2013). Molecular characterisation of *Brucella* species. Rev Sci Tech Int Off Epizoot.

[15] Jiang H, Wang H, Xu L, Hu G, Ma J, Xiao P (2013). MLVA genotyping of *Brucella melitensis* and *Brucella abortus* isolates from different animal species and humans and identification of *Brucella suis* vaccine strain S2 from cattle in China. PLoS One.

[16] Kılıç S, Ivanov IN, Durmaz R, Bayraktar MR, Ayaşlıoğlu E (2011). Multiple-locus variable-number tandem-repeat analysis genotyping of human *Brucella* isolates from Turkey. J Clin Microbiol.

[17] Georgi E, Walter MC, Pfalzgraf M-T, Northoff BH, Holdt LM, Scholz HC (2017). Whole genome sequencing of *Brucella melitensis* isolated from 57 patients in Germany reveals high diversity in strains from Middle East. PLoS One.

[18] Tan K-K, Tan Y-C, Chang L-Y, Lee KW, Norel SS, Yee W-Y, Isa MNM, Jafar FL, Hoh C-C, AbuBakar S (2015). Full genome SNP-based phylogenetic analysis reveals the origin and global spread of *Brucella melitensis*. BMC Genomics.

[19] Pisarenko SV, Kovalev DA, Khachaturova AA, Volynkina AS, Rusanova DV, Kulichenko AN (2016). Phylogeography of *Brucella melitensis* strains based on the whole-genome SNP analysis. Bacteriology.

[20] Foster JT, Beckstrom-Sternberg SM, Pearson T, Beckstrom-Sternberg JS, Chain PS, Roberto FF (2009). Whole-genome-based phylogeny and divergence of the genus *Brucella*. J Bacteriol.

[21] Bertels F, Silander OK, Pachkov M, Rainey PB, van Nimwegen E (2014). Automated reconstruction of whole genome phylogenies from short sequence reads. Mol Biol Evol.

[22] Bouckaert R, Heled J, Kühnert D, Vaughan T, Wu C-H, Xie D, Suchard MA, Rambaut A, Drummond AJ. BEAST 2: a software platform for Bayesian evolutionary analysis. PLoS Comput Biol. 10.1371/journal.pcbi.1003537. 10.1371/journal.pcbi.1003537 PMC3985171 24722319

[23] Ke Y, Yuan X, Zhen Q, Wang Y, Li T, Sun Y, et al. Genome sequence of Brucella melitensis S66, an isolate of sequence type 8, prevalent in China. J Bacteriol. 2012;194(19):5451. 10.1128/JB.01202-12 PMC3457205 22965081

[24] Ke Y, Yuan X, Wang Y, Bai Y, Xu J, Song H (2012). Genome sequences of *Brucella melitensis* 16M and its two derivatives 16M1w and 16M13w, which evolved in vivo. J Bacteriol.

[25] Özdoğan M (2011). Archaeological evidence on the westward expansion of farming communities from eastern Anatolia to the Aegean and the Balkans. Curr Anthropol.

[26] Reingruber A, Hauptmann H (2008). Die deutschen Ausgrabungen auf der Agrissa-Magula in Thessalien II. Die Agrissa Magula. Beiträge zur ur- und frühgeschichtlichen Archäologie des Mittelmeer-Kulturraums.

[27] Halphen L (1936). Les barbares: des grandes invasions aux conquêtes turques du XIe siècle, Félix Alcan.

[28] Beliaev ЕА (1965). Arabs, Islam and the Arab caliphate in the early middle ages.

[29] Cynthia C (2005). Encyclopedia of world trade: from ancient times to the present.

[30] Wurm SA, Mühlhäusler P, Tyron DT. Atlas of languages of intercultural communication in the Pacific, Asia, and the Americas. Berlin: Walter de Gruyter; 1996.

[31] Cordell LS, Lightfoot K, McManamon F, Milner G. Archaeology in America: an encyclopedia: An Encyclopedia: ABC-CLIO; Santa Barbara. 2008. p. 82–3.

[32] Allen JL. North American exploration. Lincoln: U of Nebraska Press; 2007.

[33] Kristinsson A. Expansions: competition and conquest in Europe since the bronze age. Reykjavík: Reykjavíkur Akademían; 2010.

[34] Tan K-K, Tan Y-C, Chang L-Y, Lee KW, Nore SS, Yee W-Y, Hoh CC, AbuBakar S (2017). Geographical distribution of *Brucella melitensis* inferred from *rpoB* gene variation. J Infect Dev Ctries.

[35] Andrews S (2010). FastQC: a quality control tool for high throughput sequence data.

[36] Bolger AM, Lohse M, Usadel B (2014). Trimmomatic: a flexible trimmer for Illumina sequence data. Bioinformatics.

[37] Gurevich A, Saveliev V, Vyahhi N, Tesler G (2013). QUAST: quality assessment tool for genome assemblies. Bioinformatics.

[38] Kumar S, Stecher G, Tamura K (2016). MEGA7: molecular evolutionary genetics analysis version 7.0 for bigger datasets. Mol Biol Evol.

[39] Darriba D, Taboada GL, Doallo R, Posada D (2012). jModelTest 2: more models, new heuristics and parallel computing. Nat Methods.

[40] Guindon S, Gascuel O (2003). A simple, fast and accurate method to estimate large phylogenies by maximum-likelihood. Syst Biol.

[41] Rambaut A, Suchard MA, Xie D, Drummond AJ (2014). Tracer v1.6.

[42] Rambaut A (2014). FigTree version 1.4.2.

