# Supplementary material for: Global evolution and phylogeography of Brucella melitensis strains
Source: BMC Genomics. 2018 May 10;19:353. doi: 10.1186/s12864-018-4762-2 (PMC5946514; doi:10.1186/s12864-018-4762-2)
Supplement: Supplementary file 1 — Table S1. Characteristics of genomic projects. (DOCX 17 kb) [file 12864_2018_4762_MOESM1_ESM.docx]

Table S1. Characteristics of genomic projects

| Strain | № of Contigs | N50, bp | Total length, bp | GC, % | Genes (total) | CDS (total) | Proteins | rRNAs | tRNAs | Pseudo Genes (total) |
| --- | --- | --- | --- | --- | --- | --- | --- | --- | --- | --- |
| *B. melitensis* I-136 | 46 | 155 822 | 3 285 503 | 57.20 | 3 342 | 3 286 | 2 910 | 3 | 49 | 376 |
| *B. melitensis* I-160 | 56 | 137 612 | 3 289 329 | 57.20 | 3 371 | 3 315 | 2 904 | 3 | 49 | 411 |
| *B. melitensis* I-194 | 50 | 155 784 | 3 286 088 | 57.20 | 3 364 | 3 308 | 2 910 | 3 | 49 | 398 |
| *B. melitensis* I-216 | 49 | 166 456 | 3 286 361 | 57.20 | 3 359 | 3 305 | 2 951 | 3 | 47 | 354 |
| *B. melitensis* I-280 | 49 | 166 042 | 3 286 415 | 57.20 | 3 363 | 3 307 | 2 942 | 3 | 49 | 365 |
| *B. melitensis* I-308 | 50 | 166 028 | 3 286 650 | 57.20 | 3 354 | 3 298 | 2 888 | 3 | 49 | 410 |
| *B. melitensis* I-338 | 60 | 154 969 | 3 285 883 | 57.20 | 3 368 | 3 314 | 2 963 | 3 | 47 | 351 |
| *B. melitensis* I-340 | 47 | 174 340 | 3 287 682 | 57.20 | 3 365 | 3 309 | 2 900 | 3 | 49 | 409 |
| *B. melitensis* I-349 | 37 | 201 504 | 3 282 226 | 57.20 | 3 342 | 3 286 | 3 051 | 3 | 49 | 235 |
| *B. melitensis* I-370 | 51 | 174 410 | 3 286 898 | 57.20 | 3 365 | 3 309 | 2 971 | 3 | 49 | 338 |
| *B. melitensis* KIV-L | 41 | 178 768 | 3 282 192 | 57.20 | 3 355 | 3 299 | 3 060 | 3 | 49 | 239 |
