# Supplementary material for: Global evolution and phylogeography of Brucella melitensis strains
Source: BMC Genomics. 2018 May 10;19:353. doi: 10.1186/s12864-018-4762-2 (PMC5946514; doi:10.1186/s12864-018-4762-2)
Supplement: Supplementary file 2 — Table S2. List of B. melitensis genomes used in this study. (DOCX 34 kb) [file 12864_2018_4762_MOESM2_ESM.docx]

Table S2. List of *B. melitensis* genomes used in this study

| № | Isolates | GenBank Assembly ID | Coutry | Region | Year | SNPs* |
| --- | --- | --- | --- | --- | --- | --- |
| 1 | 02-5863-1 | GCA_000480095.1 | Unknown | Unknown | - | 2303 |
| 2 | 02-7258 | GCA_000479975.1 | Unknown | Unknown | - | 2324 |
| 3 | 043 | GCA_000331655.1 | China: Shihezi Xinjiang | East Asia | - | 2404 |
| 4 | 11-1823-3434 | GCA_000480215.1 | Unknown | Unknown | - | 2316 |
| 5 | 128 | GCA_000298615.1 | China | East Asia | 1986 | 2315 |
| 6 | 133 | GCA_000298595.1 | China | East Asia | 1998 | 2313 |
| 7 | 16M | GCA_000007125.1 | USA | North America | 1952 | 0 |
| 8 | 16M_1 | GCA_000160295.1 | USA | North America | 1952 | 188 |
| 9 | 16M_2 | GCA_000250795.2 | China *(Laboratory strain)* | East Asia | 2011 | 2304 |
| 10 | 16M_3 | GCA_000740415.1 | USA | North America | 1952 | 193 |
| 11 | 16M1W | GCA_000250815.2 | China *(Laboratory strain)* | East Asia | 2011 | 2313 |
| 12 | 16M13W | GCA_000250835.2 | China *(Laboratory strain)* | East Asia | 2011 | 5819 |
| 13 | 2008724259 | GCA_001715485.1 | USA: California | North America | 2007 | 2870 |
| 14 | 2010724553 | GCA_001715465.1 | USA: California | North America | 2010 | 2334 |
| 15 | 20236 | GCA_001431745.1 | China: Inner Mongolia, Baotou city | East Asia | 2012 | 2320 |
| 16 | 548 | GCA_000591005.1 | Russia: Saratov region | Europe | 1948 | 2279 |
| 17 | 63/9 | GCA_000182235.1 | India | South Asia | 1963 | 2287 |
| 18 | 63/9_1 | GCA_000740395.1 | India | South Asia | 1963 | 2289 |
| 19 | 64/150 | GCA_000366845.1 | India | South Asia | 1964 | 2291 |
| 20 | 66/59 | GCA_000366865.1 | India | South Asia | 1966 | 2296 |
| 21 | ADMAS-G1 | GCA_000444515.1 | India | South Asia | 2013 | 206 |
| 22 | ATCC 23457 | GCA_000022625.1 | India | South Asia | 1963 | 2288 |
| 23 | B115 | GCA_000365865.1 | Malta | Europe | 1962 | 577 |
| 24 | BCB028 | GCA_000292165.2 | China | East Asia | 1956 | 2311 |
| 25 | BCB033 | GCA_000292085.2 | China | East Asia | 2006 | 2309 |
| 26 | BG2 (S27) | GCA_000370625.1 | Pakistan | Near East | 2008 | 2308 |
| 27 | BM IND1 | GCA_000685375.1 | India: North Indian plains | South Asia | 2007 | 2308 |
| 28 | Br.m-1252/10-Geo | GCA_001856295.1 | Georgia: Marneuli | Caucasus | 2010 | 2290 |
| 29 | Br.m-1268/11-Geo | GCA_001856295.1 | Georgia: Gardabani | Caucasus | 2011 | 2287 |
| 30 | Br.m-1771/12-Geo | GCA_001856285.1 | Georgia: Tbilisi | Caucasus | 2012 | 2293 |
| 31 | BRUC048 | GCA_001608355.1 | Egypt | Near East | - | 2886 |
| 32 | BRUC101 | GCA_001608425.1 | Egypt | Near East | - | 2880 |
| 33 | C-554 | GCA_000705095.1 | Russia: Republic of Dagestan | Caucasus | 2012 | 2294 |
| 34 | C-555 | GCA_000705115.1 | Russia: Republic of Dagestan | Caucasus | 2012 | 2295 |
| 35 | C-558 | GCA_000714755.1 | Russia: Republic of Dagestan | Caucasus | 2013 | 2294 |
| 36 | C-567 | GCA_001302485.1 | Russia: Republic of Kalmykia | Caucasus | 2014 | 2291 |
| 37 | C-568 | GCA_001302525.1 | Russia: Republic of Kalmykia | Caucasus | 2014 | 2295 |
| 38 | C-569 | GCA_001307425.1 | Russia: Republic of Kalmykia | Caucasus | 2014 | 2294 |
| 39 | C-570 | GCA_001302495.1 | Russia: Stavropol region | Caucasus | 2014 | 2288 |
| 40 | C-571 | GCA_001302545.1 | Russia: Stavropol region | Caucasus | 2014 | 2285 |
| 41 | C-572 | GCA_001307445.1 | Russia: Stavropol region | Caucasus | 2014 | 2286 |
| 42 | C-573 | GCA_001307475.1 | Russia: Stavropol region | Caucasus | 2014 | 2294 |
| 43 | CNGB 1076 | GCA_000366625.1 | Argentina: San Juan | South America | 2006 | 419 |
| 44 | CNGB 1120 | GCA_000366885.1 | Argentina: Buenos Aires | South America | 2006 | 420 |
| 45 | CNGB 290 | GCA_000366905.1 | Argentina: Jujuy | South America | 1996 | 424 |
| 46 | Ether | GCA_000158735.1 | Italy | Europe | 1961 | 2770 |
| 47 | Ether_1 | GCA_000740355.1 | Italy | Europe | 1963 | 2771 |
| 48 | F1/06 B10 | GCA_000370645.1 | Zimbabwe | Africa | 2006 | 1705 |
| 49 | F10/05-2 | GCA_000366925.1 | Portugal | Europe | 2005 | 607 |
| 50 | F10/06-16 | GCA_000370665.1 | Thailand | South East Asia | 2006 | 2300 |
| 51 | F15/06-7 | GCA_000365845.1 | Italy: Sicily | Europe | 2006 | 2864 |
| 52 | F2/06-6 | GCA_000366945.1 | Portugal | Europe | 2006 | 2291 |
| 53 | F3/02 | GCA_000366965.1 | Norway | Europe | 2002 | 599 |
| 54 | F5/07-239A | GCA_000366985.1 | Italy: Sicily | Europe | 2007 | 2835 |
| 55 | F6/05-6 | GCA_000367005.1 | Sudan | Africa | 2005 | 2288 |
| 56 | F8/01-155 | GCA_000370685.1 | Kosovo | Europe | 2001 | 2319 |
| 57 | F9/05 | GCA_000370705.1 | Turkey | Near East | 2005 | 2323 |
| 58 | Human/CT/US/1995 | GCA_000988815.1 | USA | North America | 1995 | 2831 |
| 59 | I-136 | GCA_002631235.1 | Russia: Republic of Chakassia | Central Asia | 1959 | 2298 |
| 60 | I-160 | GCA_002278585.1 | Russia: Republic of Tuva | Central Asia | 1961 | 2301 |
| 61 | I-194 | GCA_002278675.1 | Russia: Irkutsk region | Central Asia | 1965 | 2290 |
| 62 | I-216 | GCA_002266775.1 | Russia: Republic of Buryatia | Central Asia | 1970 | 2305 |
| 63 | I-280 | GCA_002278575.1 | Russia: Republic of Buryatia | Central Asia | 1983 | 2287 |
| 64 | I-308 | GCA_002278735.1 | Russia: Republic of Tuva | Central Asia | 1986 | 2306 |
| 65 | I-338 | GCA_002266785.1 | Russia:Novosibirsk region | Central Asia | 1993 | 2286 |
| 66 | I-340 | GCA_002278695.1 | Russia: Krasnoyarsk region | Central Asia | 1995 | 2291 |
| 67 | I-349 | GCA_002266765.1 | Russia: Republic of Tuva | Central Asia | 1999 | 2298 |
| 68 | I-370 | GCA_002278715.1 | Russia: Republic of Tuva | Central Asia | 2010 | 2295 |
| 69 | KIV-L | GCA_002266745.1 | Russia: Republic of Chechnja | Caucasus | 2014 | 2303 |
| 70 | KU/RCF-03 | GCA_001702375.1 | Kuwait | Near East | 2014 | 1771 |
| 71 | KU/RCF-31 | GCA_001702315.1 | Kuwait | Near East | 2014 | 2493 |
| 72 | KU/RCF-64 | GCA_001702335.1 | Kuwait | Near East | 2014 | 2444 |
| 73 | KU/RCF-84 | GCA_001702305.1 | Kuwait | Near East | 2014 | 2430 |
| 74 | KU/RCF-96 | GCA_001702255.1 | Kuwait | Near East | 2014 | 2376 |
| 75 | M111 | GCA_000209615.2 | China *(vaccine strain)* | East Asia | 1980s | 2323 |
| 76 | M5 | GCA_000348645.1 | China *(vaccine strain)* | East Asia | 1962 | 2320 |
| 77 | M5_1 | GCA_000209575.2 | China *(vaccine strain)* | East Asia | 1962 | 2390 |
| 78 | M5-10 | GCA_000292065.1 | China *(vaccine strain)* | East Asia | - | 2312 |
| 79 | M5-90 | GCA_000192885.1 | China *(vaccine strain)* | East Asia | - | 2317 |
| 80 | M28 | GCA_000192725.1 | China | East Asia | 1950's | 2319 |
| 81 | M28-12 | GCA_000209595.2 | China | East Asia | - | 2312 |
| 82 | MY1483/09 | GCA_000940435.1 | Malaysia | South East Asia | 2009 | 2305 |
| 83 | NI | GCA_000227645.1 | China: Inner Mongolia | East Asia | 2007 | 2403 |
| 84 | Phil1136/12 | GCA_000938035.1 | Malaysia | South East Asia | 2012 | 2308 |
| 85 | R3/07-2 | GCA_000367025.1 | Italy: Sicily | Europe | 2007 | 2849 |
| 86 | Rev.1 | GCA_000158695.1 | USA *(vaccine strain)* | North America | 1955 | 372 |
| 87 | SKN25 | GCA_001857585.1 | India | South Asia | 2015 | 2279 |
| 88 | S66 | GCA_000250775.2 | China: Jilin Province | East Asia | 2009 | 6989 |
| 89 | UK14/06 | GCA_000370725.1 | Somalia | Africa | 2006 | 1627 |
| 90 | UK19/04 | GCA_000367045.1 | Somalia | Africa | 2005 | 1625 |
| 91 | UK22/04 | GCA_000370745.1 | United Kingdom | Europe | 2004 | 1674 |
| 92 | UK22/06 | GCA_000366585.1 | Somalia | Africa | 2006 | 2306 |
| 93 | UK23/06 | GCA_000370765.1 | Somalia | Africa | 2006 | 1639 |
| 94 | UK24/06 | GCA_000370785.1 | Nigeria | Africa | 2006 | 1673 |
| 95 | UK29/05 | GCA_000370805.1 | United Kingdom | Europe | 2005 | 2271 |
| 96 | UK3/06 | GCA_000370825.1 | Cyprus | Near East | 2006 | 2283 |
| 97 | UK31/99 | GCA_000370845.1 | Egypt | Near East | 1999 | 2883 |
| 98 | UK37/05 | GCA_000370865.1 | United Kingdom | Europe | 2005 | 2316 |

* The number of SNPs found in the core genome in comparison with the reference genome *B. melitensis* 16M
