# Supplementary material for: Global evolution and phylogeography of Brucella melitensis strains
Source: BMC Genomics. 2018 May 10;19:353. doi: 10.1186/s12864-018-4762-2 (PMC5946514; doi:10.1186/s12864-018-4762-2)
Supplement: Supplementary file 3 — Table S3. Biochemical properties of isolates. (DOCX 18 kb) [file 12864_2018_4762_MOESM3_ESM.docx]

Table S3. Biochemical properties of isolates

| Strain | Location | Year | Lysis by phages | | | | Oxidase | Catalase | Urease | H2S  production | Thionin | Basic  fuchsin | Agglutination with  monospecific  antisera | | |
| --- | --- | --- | --- | --- | --- | --- | --- | --- | --- | --- | --- | --- | --- | --- | --- |
|  |  |  | **Tb** | **Wb** | **Fi** | **Bk2** |  |  |  |  |  |  | **A** | **M** | **R** |
| *B. melitensis* I-136 | Republik of Chakassia | 1959 | - | - | - | + |  | + | + | - | + | + | + | + | + |
| *B. melitensis* I-160 | Republik of Tuva | 1961 | - | - | - | + |  |  | + | - | + | + | + | - | + |
| *B. melitensis* I-194 | Irkutsk region | 1965 | - | - | - | + |  |  | + | - | + | + | + | - | + |
| *B. melitensis* I-216 | Republik of Buryatia | 1970 | - | - | - | + |  |  | + | - | + | + | + | - | + |
| *B. melitensis* I-280 | Republik of Buryatia | 1983 | - | - | - | + |  |  | + | - | + | + | - | + | + |
| *B. melitensis* I-308 | Republik of Tuva | 1986 | - | - | - | + |  |  | + | - | + | + | - | + | + |
| *B. melitensis* I-338 | Novosibirsk region | 1993 | - | - | - | + |  |  | + | - | + | + | + | + | + |
| *B. melitensis* I-340 | Krasnoyarsk region | 1995 | - | - | - | + |  |  | + | - | + | + | - | + | + |
| *B. melitensis* I-349 | Republik of Tuva | 1999 | - |  |  |  |  |  | + | - |  |  | + | + | + |
| *B. melitensis* I-370 | Republik of Tuva | 2010 | - | - | - | + |  |  |  | - | + | + | - | + | + |
| *B. melitensis* KIV-L | Republic of Chechnya | 2014 | - | + | - | + | + | + | + | - | + | + | + | + | + |
