# Supplementary material for: Global evolution and phylogeography of Brucella melitensis strains
Source: BMC Genomics. 2018 May 10;19:353. doi: 10.1186/s12864-018-4762-2 (PMC5946514; doi:10.1186/s12864-018-4762-2)
Supplement: Supplementary file 4 — Table S4. Antibiotic susceptibility testing. (DOCX 16 kb) [file 12864_2018_4762_MOESM4_ESM.docx]

Table S4. Antibiotic susceptibility testing

| Strain | Gentamicin | Amikacin | Kanamycin | Streptomycin | Tetracycline | Ciprofloxacin | Ofloxacin | Pefloxacin | Levofloxacin | Rifampicin |
| --- | --- | --- | --- | --- | --- | --- | --- | --- | --- | --- |
| *B. melitensis* I-136 | ≥20 | ≥20 | ≥20 | ≥20 | ≥25 | ≥30 | ≥25 | ≥25 | ≥25 | ≥15 |
| *B. melitensis* I-160 | ≥20 | ≥20 | ≥20 | ≥20 | ≥25 | ≥30 | ≥25 | ≥25 | ≥25 | ≥15 |
| *B. melitensis* I-194 | ≥20 | ≥20 | ≥20 | ≥20 | ≥25 | ≥30 | ≥25 | ≥25 | ≥25 | ≥15 |
| *B. melitensis* I-216 | ≥20 | ≥20 | ≥20 | ≥20 | ≥25 | ≥30 | ≥25 | ≥25 | ≥25 | ≥15 |
| *B. melitensis* I-280 | ≥20 | ≥20 | ≥20 | ≥20 | ≥25 | ≥30 | ≥25 | ≥25 | ≥25 | ≥15 |
| *B. melitensis* I-308 | ≥20 | ≥20 | ≥20 | ≥20 | ≥25 | ≥30 | ≥25 | ≥25 | ≥25 | ≥15 |
| *B. melitensis* I-338 | ≥20 | ≥20 | ≥20 | ≥20 | ≥25 | ≥30 | ≥25 |  | ≥25 | ≥15 |
| *B. melitensis* I-340 | ≥20 | ≥20 | ≥20 | ≥20 | ≥25 | 25-30 | ≥25 | ≥20 | ≥25 | 10-15 |
| *B. melitensis* I-349 | ≥20 | ≥20 | ≥20 | ≥20 | ≥25 | ≥30 | ≥25 | ≥25 | ≥25 | ≥15 |
| *B. melitensis* I-370 | ≥20 | ≥20 | ≥20 | ≥20 | ≥25 | ≥30 | ≥25 |  | ≥25 | ≥15 |
| *B. melitensis* KIV-L | ≥20 | ≥20 | ≥20 | ≥20 | ≥25 | ≥30 | ≥25 | ≥25 | ≥25 | ≥15 |
